# Maladie de Kaposi classique avec atteinte du système musculo-squelettique: un nouveau cas

**DOI:** 10.11604/pamj.2014.17.17.2804

**Published:** 2014-01-15

**Authors:** Meryem Soughi, Fatima Zahra Mernissi

**Affiliations:** 1 CHU Hassan II, Fès, Maroc

**Keywords:** Maladie de Kaposi, système musculo-squelettique, Kaposi disease, musculoskeletal system

## Image en medicine

**Figure 1:**
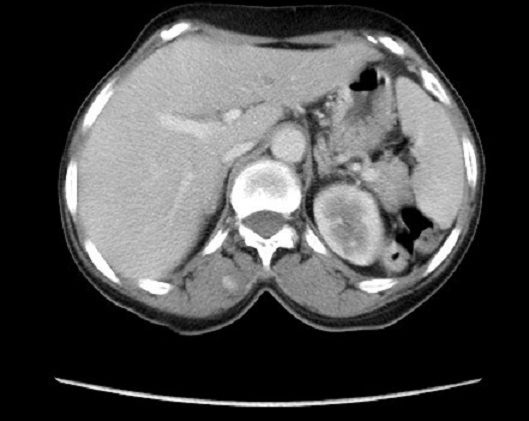
Coupe axiale montrant une lésion musculaire para vertébrale droite de 3 cm prenant fortement le contraste

La maladie de Kaposi est une maladie angioproliferative multifocale, dont les localisations cutanéo-muqueuses sont les plus fréquentes. Nous en rapportons un cas avec une atteinte musculo squelettique. Patiente de 55 ans, présente depuis un an de multiples nodules angiomateux confluents en des tumeurs ulcéro bourgeonnantes sur un placard hyperpigmenté et oedème de la jambe gauche, associés à des macules angiomateuses de la plante du pied droit. La biopsie cutanée était en faveur d'une maladie de kaposi, la sérologie VIH était négative. Le bilan lésionnel objectivait une lyse du premier et du deuxième métatarsien, des micronodules parenchymateux pulmonaires bilatéraux, des adénopathies pré trachéales, et une lésion musculaire para vertébrale de 3 cm prenant fortement le contraste (Figure 1). L'atteinte du système musculo-squelettique dans la maladie de Kaposi est très rare, seulement 66 cas publiés dans la littérature, dont 3 uniquement comprenaient une atteinte musculaire, avec une sérologie VIH positive. Les lésions osseuses étaient les plus fréquentes. Les patients étaient rarement asymptomatiques, presque tous les patients avaient des lésions concomitantes non osseuses. Chez notre patiente, il s'agit d'une maladie de Kaposi classique avec une atteinte multifocale: cutanée, pulmonaire, ganglionnaire, osseuse par contigüité et musculaire para vertébrale sans atteinte cutanée en regard, ce qui fait la particularité de notre observation. Un traitement à base d'une chimiothérapie était instauré avec une bonne réponse. L'atteinte du système musculo squelettique bien qu'elle est rarissime, son exploration n'est pas systématique tant qu'elle est asymptomatique, sa prise en charge rejoint celle de la maladie de kaposi.

